# Current Trends in AI Gait Analysis for the Detection and Assessment of Parkinson’s Disease Severity: Systematic Review and Meta-Analysis of Performance Using Logit Transformation

**DOI:** 10.3390/healthcare14131820

**Published:** 2026-06-23

**Authors:** Philippe Gorce, Julien Jacquier-Bret

**Affiliations:** 1 University of Toulon, CS 60584, CEDEX 9, 83041 Toulon, France; gorce@univ-tln.fr; 2 International Institute of Biomechanics and Occupational Ergonomics, Avenue du Docteur Marcel Armanet, CS 10121, 83418 Hyères, France

**Keywords:** artificial intelligence, Parkinson, machine learning, deep learning, accuracy, abnormal gait, VGRF, severity, H&Y scale, PhysioNet

## Abstract

Background/Objectives: Artificial intelligence (AI) offers a promising approach for detecting and classifying symptom severity in patients with Parkinson’s disease (PD). The objective was to provide an overview of AI methods performance used for this classification through a systematic review and meta-analysis conducted in accordance with the PRISMA (Preferred Reporting Items for Systematic Reviews and Meta-Analyses) guidelines. Methods: The Google Scholar, IEEE Xplore, PubMed/MedLine, and ScienceDirect databases were searched for the period 2015–2025. The studies included were original, peer-reviewed studies written in English that addressed an AI method based on machine learning (ML) or deep learning (DL) for the classification of PD patients. The dataset used had to be “Gait in Parkinson’s Disease,” in which the severity of disease symptoms was assessed using the Hoehn and Yahr (H&Y) scale. Studies had to report at least one of the five performance metrics: accuracy, sensitivity, specificity, precision, and F1 score. Two reviewers independently selected articles, assessed the risk of bias using PROBAST (Prediction Model Study Risk of Bias Assessment Tool), and extracted data. The logit-transformed values were pooled separately by performance metrics and by severity level using a random-effects model. Cochran’s Q test, the I^2^ statistic, and inter-study variability (τ^2^), computed using the generalized inverse variance method with the restricted maximum likelihood model, were used to assess heterogeneity. Forest plots with 95% confidence intervals were used to present the results. Possible causes of heterogeneity were explored using a subgroup analysis (ML vs. DL) and a sensitivity analysis. Finally, publication bias (Egger’s test) and the certainty of the evidence (using GRADE—Grading of Recommendations Assessment, Development, and Evaluation) were assessed to verify the generalizability of the results. Results: Among the 257 unique records, 12 studies were included. The methods demonstrated very high overall performance (>92%): accuracy (96.4%, 95% CI: 95.9–96.9%), specificity (97.7%, 95% CI: 97.3–98.1%), sensitivity (94.0%, 95% CI: 92.7–95.2%), precision (93.4%, 95% CI: 92.0–94.6%), F1 score (92.1%, 95% CI: 90.6–93.4%). Accuracy, specificity, and precision were high for all H&Y levels. However, the more advanced the symptoms, the lower the sensitivity (97.3% for H&Y0 vs. 92.1% for H&Y3). ML models achieved the best results for classifying healthy patients (H&Y0: 95.7% to 98.2%), while DL approaches performed better for classifying higher severity levels (>92%). Heterogeneity and inter-study variability were moderate (I^2^: 40–50% and τ^2^: 0.3–0.4) for precision and F1 score, and high (I^2^ > 90% and τ^2^ > 0.6) for accuracy, specificity, and sensitivity. The GRADE analysis revealed low-quality evidence for precision and F1 score and very-low quality for accuracy, specificity, and sensitivity. Conclusions: Thus, AI-based wearable gait assessment devices show great promise in terms of aiding clinical decision-making and treatment personalization. However, further research using a rigorous methodology (PROBAST) is needed to ensure the generalizability of the results and the clinical viability of the proposed solutions.

## 1. Introduction

Parkinson’s disease (PD) is the second most common neurodegenerative disease in the world after Alzheimer’s disease [1,2]. According to the Global Burden of Disease study, it is the neurological disease with the fastest growing prevalence [3]. The number of people affected has risen from 3.15 million cases in 1990 to 11.77 million in 2021 [4], and a global projection estimates that this number will increase to 25.2 million by 2050 [5]. The main motor symptoms that characterize PD are bradykinesia, resting tremors, rigidity, and abnormalities in posture and gait [6,7]. However, PD is not limited to motor symptoms. Patients frequently exhibit neuropsychiatric manifestations such as depression, anxiety, apathy, or cognitive impairment [8]. Depression is one of the most common non-motor symptoms (30–50% of patients) and is associated with worsening functional disability, including impaired gait characteristics (speed, cadence, stride length), as well as an increased risk of postural instability and freezing of gait [9]. Patients therefore resort to pharmacological treatment of depressive disorder to limit its impact on motor symptoms [10]. As this disease is progressive, the severity of symptoms varies from one individual to another. This leads to a deterioration in their quality of life, initially manifesting as difficulties in performing everyday activities such as dressing and eating, and eventually leading to wheelchairs or bedridden status. In order to optimize therapeutic intervention, the development of effective early diagnosis systems has become an important area of research.

Among the various approaches, gait analysis has developed significantly with advances in technology and computing. Gait analysis can be performed using non-wearable sensors such as high-speed cameras, optoelectronic systems, or force platforms that can quantify a large number of biomechanical parameters. In addition, wearable solutions have been greatly improved and democratized over the past 20 years. These devices allow continuous analysis of gait. These include inertial measurement units (IMUs), electromyography (EMG) sensors, plantar pressure sensors, and physiological sensors (heart rate, temperature, etc.). These methods provide essential information, particularly on gait symmetry, spatio-temporal or frequency parameters, and joint movements [11,12], in order to qualify and quantify the severity of PD patients’ symptoms [13]. One of the major challenges is managing the large amount of heterogeneous data and the complexity of the correlations between parameters in order to make an accurate diagnosis. In this context, artificial intelligence has shown significant potential in helping to diagnose PD and classify its different stages based on the motor symptoms [14,15]. Two approaches are commonly used. Traditional machine learning (ML) approaches rely on manual extraction of predefined characteristics of gait to perform classification. ML methods incorporate several algorithms, among which support vector machines (SVM), decision trees (DT), the k-nearest neighbors (KNN) method, and random forests (RF) have been the most widely used [16,17]. More recently, deep learning has been applied to gait analysis. The main advantage of this approach is the automatic extraction of features using convolutional neural networks (CNN) or recurrent neural networks (RNN) such as LSTM (Long Short-Term Memory), bi-LSTM, or GRU (Gated Recurrent Unit) [18,19]. These methods have been used both for binary classification of PD vs. healthy subjects and for multiclass classification of severity [20,21].

One of the important aspects in developing a method for detecting and classifying PD is its performance. This is achieved using a confusion matrix that evaluates the number of true and false positives and negatives required to compute the performance metrics. The most commonly used metrics are accuracy, precision, specificity, sensitivity, and F1 score [22,23]. Numerous ML or DL solutions have demonstrated high performance levels exceeding 90% or even 95% accuracy in detecting PD patients [24,25]. However, the populations used, the age of the subjects, the severity, number of patients per level, etc., are all factors that influence these results. In particular, it has been shown that the database used to train and test the algorithms has a direct impact on performance [26]. It can therefore be difficult to determine the most effective methods. Having an overview of the performance of existing AI solutions for the detection and classification of the severity of Parkinson’s disease patients in similar conditions would be a major asset for early management of the disease and thus delaying the loss of autonomy.

The objective of this study was to propose a systematic review with meta-analysis to evaluate the performance of artificial intelligence methods for the detection and classification of PD patients in a controlled setting. The hypothesis was that the use of a common dataset would reduce the variability in reported performance and thus enable the identification of ML or DL methods with the most optimal ability to detect disease severity. To achieve this objective, this study proposes an analysis of detection performance according to the severity stage of symptoms obtained from the most widely used classification, i.e., the Hoehn and Yahr (H&Y) Scale [27] and the AI method (ML vs. DL).

## 2. Related Work

The progression of Parkinson’s disease is typically assessed using two clinical rating scales. The first is the Movement Disorder Society’s Unified Parkinson’s Disease Rating Scale (MDS-UPDRS) [28]. This is a comprehensive tool for assessing motor and non-motor symptoms in everyday life. Esan et al. [29] used this scale to develop an interpretable machine learning model for early and accurate prediction of Parkinson’s disease. Dennis et al. [30] conducted a systematic review on the subject of detection, reporting different methods with their performance for different MSD-UPDRS levels. The second is the Hoehn and Yahr (H&Y) scale, which focuses more on posture and mobility. The modified 7-level version (1, 1.5, 2, 2.5, 3, 4, 5) considers laterality and severity of impairment to classify a patient from a situation of almost complete independence to the use of a wheelchair or complete bedridden status [27]. It remains a universally recognized assessment scale for its effectiveness in providing comprehensive validation of disease severity based on functional disability and is used in numerous studies. Li et al. [31] proposed a machine learning model and evaluated its performance based on the PD severity. Mittal et al. [32] tested the performance of several classification algorithms (Decision Tree, Random Forest, Extreme Gradient Boost, and Light Gradient Boosting) to predict severity considering 3 levels of the H&Y scale: 2, 2.5, and 3.

Since the validation and accuracy of the proposed methods are key issues in AI, the authors systematically assess the performance. Several datasets were used in the literature. Prabhu et al. [33] used the Neuro-Degenerative Disease Gait Dynamics (NDDGD) dataset with 13 healthy subjects and 13 subjects with Parkinson’s disease. The authors reported an accuracy of 94.44%, a specificity of 90.00%, and a sensitivity of 91.00% for binary classification with their probabilistic neural networks. Many authors have used vertical ground reaction force from the Gait in Parkinson’s Disease dataset from PhysioNet. It consists of 93 people affected with PD and 73 healthy controls, including contributions from Yogev et al. [34], Hausdorff et al. [35], and Frenkel-Toledo et al. [36]. Torghabeh et al. [37] used their DL method based on a bi-LSTM to classify subjects with a performance for accuracy, specificity, sensitivity, precision, and F1-score greater than 95%. Zeng et al. [38] used different versions of neural networks to perform classification with similar performance. Other authors evaluated the performance of their method using custom datasets. Archila et al. [39] tested their method based on video analysis and fusion of deep or kinematic features on 13 healthy subjects and 13 subjects with Parkinson’s disease, with performance ranging from 85% to 100% for accuracy, specificity, sensitivity, precision, and F1-score. Buongiorno et al. [40] used a Kinect to classify 14 healthy subjects and 16 PD subjects using an SVM with accuracy, specificity, and sensitivity values between 70% and 95%.

Several systematic reviews have been conducted on the PD detection using gait analysis. Some authors have compiled a state-of-the-art review considering only one method. di Biase et al. [41] conducted a systematic review of ML methods for automated PD diagnosis using wearable sensors. Based on 51 studies, the authors concluded that SVM were the prevalent algorithm and suggested promising accuracy with complex algorithms such as RF, SVM, and KNN. Franco et al. [42] studied the role of DL through a systematic review including 25 articles using both wearable and non-wearable sensors. The authors concluded that DL enables the prediction of PD and disease severity. However, the choice of the best deep neural network architecture may depend on the size and complexity of the dataset and the specific aims of the research or application. Guo et al. [43] conducted a survey and summarized the different sensors and the two main methods (ML and DL) used in AI, as did Rabie et al. [44] in a review. By reporting the performance data from the studies included in their work, these authors demonstrated promising ML and DL solutions for improving diagnostic accuracy.

In light of this work, the major contributions of this study would be:

- To propose a methodical synthesis and systematic review of AI methodologies for PD severity detection based on ML and DL models by following the PRISMA guidelines and applying the logit transformation.
- To present and compare the performance parameters of different methods according to severity level (H&Y0, 2, 2.5, and 3) and the AI method (ML, DL) used to consider a unique dataset to limit heterogeneity, data disparity, and evaluation conditions.

## 3. Materials and Methods

### 3.1. Study Design

The present study is a systematic review and meta-analysis following the PRISMA guidelines (Preferred Reporting Items for Systematic reviews and Meta-Analyses) [45,46,47]. The aim was to study the performance of AI algorithms for detecting individuals with PD and their ability to identify the level of severity using H&Y scale, based on gait analysis. The studies had to be original and include at least one performance metric (e.g., accuracy, sensitivity, specificity, precision, or F1 score).

### 3.2. Severity of Parkinson’s Disease and Dataset

To limit heterogeneity and disparity in data from different assessment conditions, a single dataset was used in this analysis: the Gait in Parkinson’s Disease dataset from PhysioNet. It contains the vertical ground reaction force signal (100 Hz) during walking from eight resistive force sensors attached to the insole of each foot for 93 PD patients and 73 healthy control subjects. Three severity levels based on the H&Y scale were considered in this dataset: 2 (bilateral involvement without impairment of balance), 2.5 (mild bilateral disease with recovery on pull test and impairment of balance), and 3 (mild to moderate bilateral disease with impaired postural reflexes) [27]. Three authors contributed to the development of this dataset: Yogev et al. [34], Hausdorff et al. [35], and Frenkel-Toledo et al. [36], designated Ga, Ju, and Si, respectively. Table 1 details the content of these three parts.

### 3.3. Search Strategy

To achieve the objective, a search was conducted between 7 and 30 January 2026, in Google Scholar, IEEE Xplore, PubMed/MedLine, and ScienceDirect using a detailed list of keywords associated with the logical operators AND and OR. The search was limited to articles published in the last decade, i.e., from 2015 to 2025 inclusive. Table 2 details the search strategy. Due to differences between search engines, the list of keywords for ScienceDirect had to be slightly modified.

### 3.4. Selection Criteria

The inclusion of studies was based on several criteria. To be selected, a study had to present an AI method for detecting Parkinson’s disease based on an ML or DL approach. It had to present the values of one or more performance parameters for each severity level from the Ga, Ju, and Si datasets. The performance parameters selected were accuracy, precision, specificity, sensitivity, and F1 score. Only original, published, peer-reviewed studies were considered.

The following exclusion criteria were applied: (1) the study was a conference paper, book or chapter, review, report, case study, or case report; (2) the study was not written in English; (3) the study had not been peer-reviewed; (4) the datasets were not exclusively Ga, Ju, Si; (5) performance parameters were not available by severity level (H&Y scale).

### 3.5. Risk of Bias Assessment

The risk of bias in the included studies was assessed using the PROBAST tool (Prediction Model Study Risk of Bias Assessment Tool) [48]. The PROBAST tool contains 20 items grouped into four domains: participants (2 items), predictors (3 items), outcomes (6 items), and analysis (9 items). The assessment was conducted in three stages. First, each item was rated with one of the following responses: “yes”, “probably yes”, “probably no”, “no”, or “information missing”. The wording was designed so that “yes” indicated the absence of bias. Second, the risk of bias in each of the four domains was classified according to the responses associated with the corresponding items: “low” if all responses were “yes” or “probably yes”, “high” if at least one item received a “no” or “probably no” response, and “uncertain” if at least one response was “information missing” and the other responses were “yes” or “probably yes”. Third, the overall risk of bias was determined based on the following rules: “low risk of bias” if all four domains were at low risk of bias, “uncertain risk of bias” if at least one domain was at uncertain risk of bias and all others were at low risk, and “high risk of bias” if at least one domain was at high risk of bias. Since model validation is essential, its absence led to a “high risk of bias” assessment. The entire analysis was conducted independently by two reviewers (J.J.B. and P.G.). Any discrepancies were resolved by consensus. A traffic light diagram was used to present the results [49].

### 3.6. Data Extraction and Classification

First, the name of the first author and the year of publication were noted. Then, for each study, the AI method (ML or DL), the nature of the algorithms used (e.g., CNN, SVM, KNN, LSTM, DT, etc.) and the values of the five performance parameters (accuracy, precision, sensitivity, specificity, and F1 score) were extracted. These parameters are computed from the four information contained in the confusion matrix: true positives (TP, correct positive predictions), true negatives (TN, correct negative predictions), false positives (FP, incorrect positive predictions), and false negatives (FN, incorrect negative predictions). The formulas used are as follows: accuracy = (TP + TN)/(TP + TN + FP + FN), sensitivity = TP/(TP + FN), specificity = TN/(FP + TN), precision = TP/(TP + FP), F1 score = 2 × (sensitivity × precision)/(sensitivity + precision).

### 3.7. Statistical Analysis

To compare the methods, data on precision, specificity, sensitivity, accuracy, and F1 score were pooled by severity level (scores of 2, 2.5, and 3 on the H&Y scale for PD and H&Y0 for healthy subjects), and then by AI method (ML vs. DL). Since the performance metrics are bounded indicators with an asymmetric sampling distribution, a logit transformation was first applied to stabilize their variance [50]. The formula was as follows:

(1) $$logit\left(p\right)=ln\left(\frac{p}{1-p}\right)=x$$

where *p* is performance (comprise entre 0 et 1) and *ln* the is natural logarithm.

The corresponding standard error (*SE*) was computed using the formula:

(2) $$SE\left(logit\left(p\right)\right)=\sqrt{\frac{1}{p}+\frac{1}{n-p}}$$

where *n* corresponds to the number of subjects.

If *p* is equal to 0 or 1, the logit transformation and the calculation of the associated standard error become mathematically impossible. In accordance with international methodological recommendations [51,52], a Haldane–Anscombe continuity correction (*p*_(*HA*)_) was applied to recompute *p* by adding 0.5 to the sample sizes in order to enable the logit transformation and its variance to be computed [53,54] as follows:

(3) $$p_{\left(HA\right)}=\frac{p+0.5}{n+1}$$

Meta-analyses were conducted separately for each performance parameter. The logit-transformed values were pooled across all levels and for each level of H&Y scale using a random-effects model due to high heterogeneity in performance. Data heterogeneity was assessed using Cochran’s Q-test (with a significance level set at 10%) and the I^2^ statistic [55]. I^2^ quantifies the degree of variability (expressed as a percentage) attributable to heterogeneity across four levels: low heterogeneity (0–40%), moderate heterogeneity (30–60%), substantial heterogeneity (50–90%), and high heterogeneity (75–100%) [55]. In addition, inter-study variability (τ^2^) was estimated using the inverse variance method with the restricted maximum likelihood (REML) model. For each analysis, the results were synthesized using a forest plot that included the 95% confidence intervals computed using the standard method and the weight of each study. All results were compiled into a summary table.

The analysis was expanded by exploring the causes of heterogeneity. A subgroup analysis was conducted to assess the effect of the AI method used, i.e., ML vs. DL methods. Finally, the robustness of the meta-analysis results was assessed using a sensitivity analysis with a leave-one-out method, and Egger’s test [56] was used to determine publication bias (significance threshold set at 5%).

To facilitate interpretation, all results were back-transformed to the original scale using:

(4) $$p=antilogit\left(x\right)=\frac{e^{x}}{1+e^{x}}$$

where *x* is the value on the logit scale (Equation (1)).

All analyses were performed with JASP software (JASP Team, v0.95.4.0, Amsterdam, The Netherlands). The significance threshold was set at 5%.

### 3.8. Certainty Assessment

The GRADE (Grade of Recommendations Assessment, Development and Evaluation) method [57] was applied to assess the quality of the evidence for each performance parameter. The GRADE method comprises the following five domains: risk of bias, indirectness of evidence, inconsistency of results, imprecision of results, and publication bias. These five GRADE domains were assessed separately by two reviewers (P.G. and J.J.B.). Based on this assessment, an overall GRADE rating was assigned according to a four-level scale: high, moderate, low, and very low. The results obtained by each reviewer were compared and discussed to validate the final rating.

### 3.9. Registration

The protocol was registered in PROSPERO (CRD420261337729).

## 4. Results

### 4.1. Search Results

**Figure 1:**
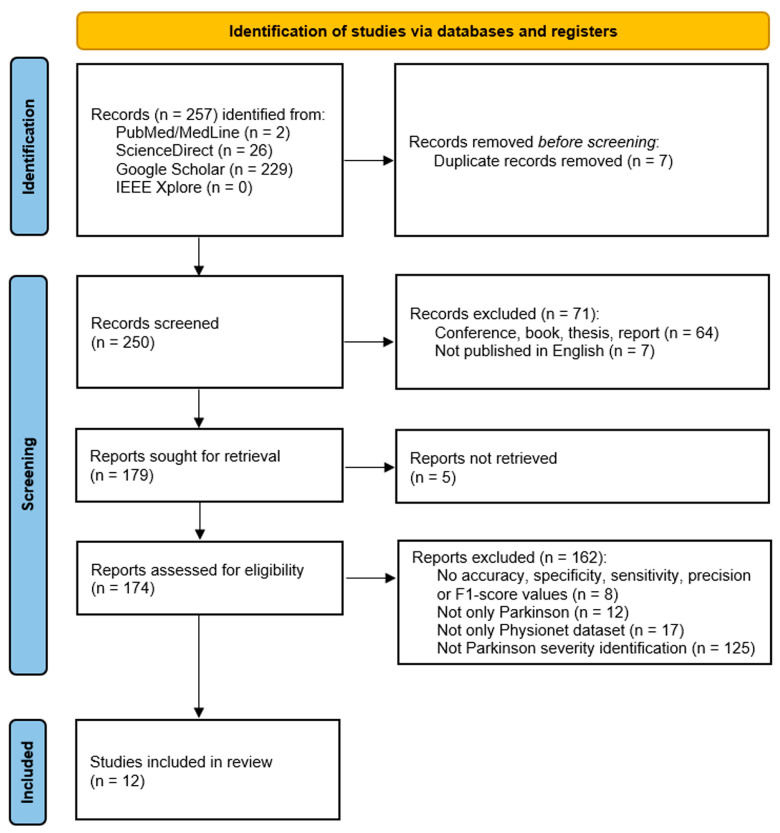
PRISMA flow diagram.

A total of 257 articles were identified during the search of the four databases. Seven duplicates were removed. Seventy-one articles were excluded from the 250 unique articles, either because they were not written in English or because they were not original peer-reviewed articles (conference papers, theses, reports, books, etc.). From the remaining 179 articles, 162 were excluded after reading the full text for the following reasons: the dataset used was not only from PhysioNet (Ga, Ju, Si), the population included pathologies other than Parkinson’s disease, the performance parameters of the algorithms were not reported, or the detection of the Parkinson severity level was not investigated. Five additional articles were excluded because the full text could not be retrieved. Ultimately, the search included 12 articles. The entire process is illustrated in Figure 1.

### 4.2. Study Characteristics

Table 3 details the information relating to the studies included, specifying the AI methods and algorithms used, the number of methods tested, and the parameters used to evaluate the performance of each method. The 12 studies were spread across three countries: Canada (1 study) [58], China (2 studies) [59,60], India (8 studies) [14,32,61,62,63,64,65,66], and the USA (1 study) [67]. Seven studies used an ML method [14,32,59,60,61,64,65] and five used a DL approach [58,62,63,66,67]. For the ML approaches, 10 different algorithms were used: DT, SVM, KNN, RF, AdaBoost (Adaptive Boosting), BC (Bayes Classifier), EC (Ensemble classifier), LightGBM (Light Gradient-Boosting Machine), NB (Naïve Bayes Classifier), and XGBoost (eXtreme Gradient Boosting). For the DL methods, only three algorithms were tested: CNN, GRU, and LSTM.

Regarding performance evaluation, four studies used three parameters [58,60,61,65], four studies used four parameters [32,59,62,67], and four studies used all five parameters considered [14,63,64,66]. The most commonly reported performance parameter was sensitivity (reported by all 12 studies included), followed by accuracy and F1-score (10 studies) and specificity and precision (8 studies). Taking into account all the methods tested, this study included 176 different algorithms distributed as follows for the five parameters: accuracy (165 values), specificity (148 values), sensitivity (176 values), precision (72 values), and F1-score (100 values).

### 4.3. Risk of Bias

**Figure 2:**
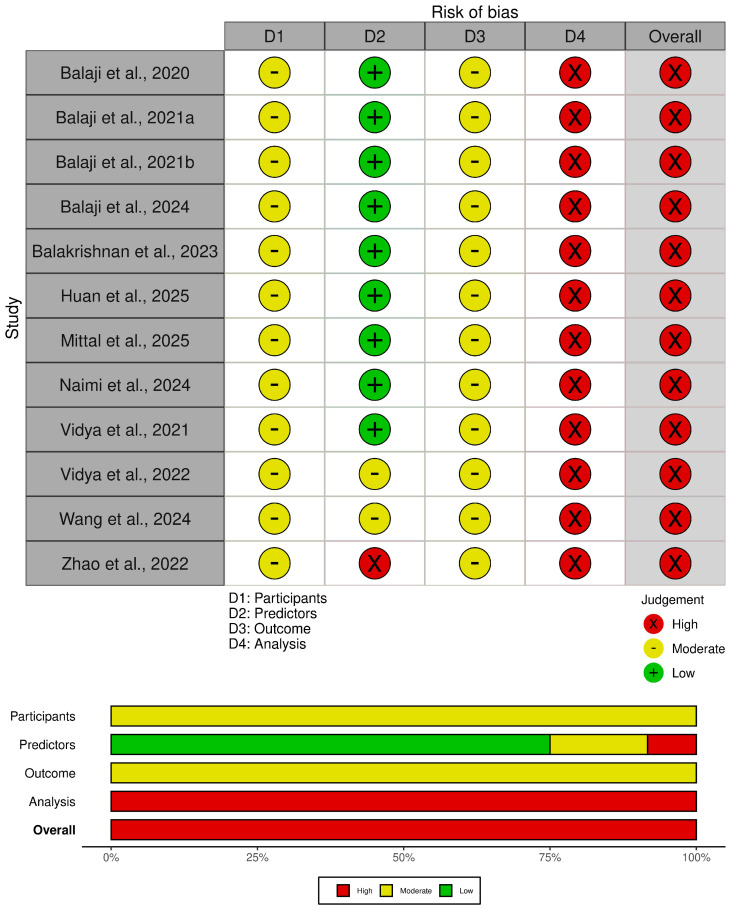
Traffic-light plot: summary of the risk of bias. References [14,32,58,59,60,61,62,63,64,65,66,67].

All studies presented a high risk of bias, due to domain “Outcome” and “Analysis”, in which either the training phase was not clearly detailed, or the distribution of data between the training, validation, and testing phases was not clearly mentioned, and because external validation was not carried out (Figure 2).

### 4.4. Performance Detection by H&Y Score

Accuracy and specificity both exceeded 95% for all classes. Sensitivity, precision, and F1 score ranged between 90% and 95%, with the best performance for sensitivity in class H&Y0 (97.3%) and the lowest for the F1 score in class H&Y3 (85.8%).

For accuracy, the data are relatively homogeneous (1.4% difference) with a slight advantage for the classification of the two extreme classes (H&Y0 and H&Y3). A similar observation was made for sensitivity. The maximum difference was 1.1%, with the highest value for class H&Y3 (98.2%). Greater variation was observed for the other three parameters, with the best performance achieved for the H&Y0 class. For sensitivity, the maximum difference was 7.3% between H&Y0 (97.3%) and H&Y2.5 (90.0%). For precision, the difference was 3.1% between H&Y0 (95.1%) and H&Y3 (92.0%). The largest difference (9.1%) was observed for the F1 score between H&Y0 (94.9%) and H&Y3 (85.8%).

The meta-analysis revealed substantial heterogeneity (I^2^ > 50%) and inter-study variance (τ^2^ > 0.40) for the majority of performance parameters. Table 4 presents all these values in detail, as well as each performance with its 95% confidence interval. All forest plots displaying the results in detail are available in Supplementary Files (Figures S1–S5).

### 4.5. Performance Using Only the Best AI Methods for Each Study

When considering all the proposed algorithms, there is a significant imbalance in the data from the 12 included studies. Indeed, some studies tested only a single method, while others tested a larger number. When multiple algorithms are tested, they are derived from the same participants and the same dataset and are therefore not statistically independent. To avoid an artificial increase in sample size that could skew the pooled estimates, an in-depth analysis was conducted by selecting only the best AI method from each of the 12 included studies. Thus, the data contained only one performance value per reported performance parameter for each of the 4 levels of the H&Y scale (i.e., 4 data rows per study). Table 5 presents the differences observed between the results obtained previously (where all results were considered) and those obtained using only the best AI methods. Across all H&Y levels, A low variation in pooled performance was observed (increases ranging from +0.48% to +1.64%). By H&Y level, a similar result was observed: an increase ranging from 0.26% to 1.18% for H&Y0, from 0.45% to 1.49% for H&Y2, and from −0.05% to 2.40% for H&Y2.5. Only the precision and F1 score values for H&Y3 showed a significant increase of 3.97% and 6.96%, respectively. Regarding heterogeneity, a decrease in I^2^ values was observed for a large proportion of the pooled performances. However, for some of them, it remained constant or even higher (e.g., sensitivity for H&Y2 and 3, precision for all classes and H&Y0, or F1 score for H&Y0 and 2). Despite the observed decrease, a large proportion of I^2^ values remained above 50%, reflecting significant variability. Appendix A contains details of all performance values obtained from the best AI method in each study used to construct Table 5.

### 4.6. Comparison of AI Methods by H&Y Score

Table 6 presents the pooled values, the 95% confidence intervals, and the heterogeneity statistics (I^2^ and τ^2^) for the five performance parameters for each level of the H&Y scale, separately for ML and DL methods. The values obtained for DL ranged from 92.3% to 99.3%, and for ML from 77.4% to 98.2%.

The results showed relative homogeneity (I^2^ < 20% and τ^2^ < 0.05) in the performance of DL algorithms for accuracy (H&Y2 and H&Y2.5 classes), specificity across all classes, as well as sensitivity (H&Y2.5 class), precision and F1 score for H&Y2.5 and H&Y 3. Consistency among ML algorithms was observed only for precision in the H&Y2.5 class.

**Figure 3:**
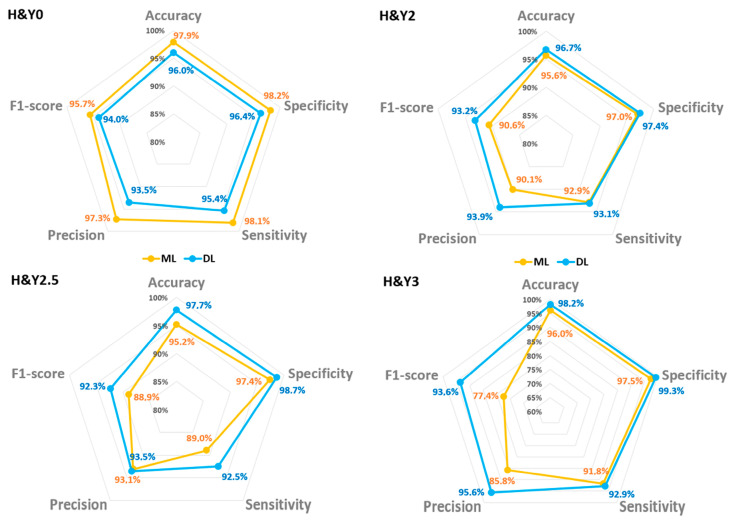
Radar chart of performance parameters by H&Y score for ML and DL methods.

In terms of performance, the ML models delivered the best overall results for the H&Y0 class. The values ranged from 95.7% (F1-score) to 98.2% (specificity), while DL performance ranged from 93.5% (precision) to 96.4% (specificity), as shown in Figure 3. Conversely, for the other three classes, the DL approaches outperformed the ML approaches. Performance remained consistently high, with values above 92% (reaching a maximum of 99.3% for the specificity of the H&Y3 class). In comparison, ML models showed more heterogeneous and overall lower performance, with an F1-score below 80% for the H&Y3 class and two metrics ranging between 80% and 90% (sensitivity and F1 score for H&Y3). However, similar values were observed for specificity and sensitivity for the H&Y2 class, precision for the H&Y2.5 class, and sensitivity for the H&Y3 class.

These results suggest better robustness of DL approaches for distinguishing advanced stages of the disease, while ML methods appear to retain an advantage for identifying H&Y0 subjects.

### 4.7. Sensitivity Analysis

The sensitivity analysis was conducted by successively excluding studies one by one. For accuracy, specificity, and precision, the largest difference from the pooled value across all studies was less than 1%. For sensitivity and F1 score, the maximum differences were 1.43% and 1.09%, respectively. The results are detailed in Table 7.

### 4.8. Publication Bias

Visual inspection of the funnel plots suggested asymmetry for all performance parameters and all H&Y levels. Egger’s regression test was statistically significant (*p* < 0.05), indicating a possible publication bias or small-study effect. The studies with the best performance had a higher standard error. However, this asymmetry could also be related to the high inter-study heterogeneity and the methodological differences observed between studies. Sensitivity analyses showed that the pooled estimates remained relatively stable despite this potential bias.

### 4.9. Level of Evidence

The initial level associated with the diagnostic test accuracy studies was “high,” but it had to be downgraded several times due to serious risks observed across various GRADE domains. First, the level was downgraded once due to a significant Egger’s test for all parameters, indicating the presence of small-study effects or publication bias. A second downgrade was performed due to the high risk of bias observed in the PROBAST analysis for all included studies. For accuracy, sensitivity, and specificity, inconsistency was observed due to the high heterogeneity of the results (I^2^ > 50%), which led to a third downgrade for these three performance parameters. Thus, the GRADE analysis identified a low level of evidence for precision and F1-score and a very low level of evidence for accuracy, sensitivity, and specificity. Table 8 details the GRADE analysis.

## 5. Discussion

The objective of this systematic review was to provide an overview of the PD detection and the classification of its severity using AI. Knowledge of the performance of the proposed methods is essential for early diagnosis, thereby enabling the progression of symptoms to be controlled and the loss of autonomy in patients to be better managed. The analysis includes traditional ML and DL methods to detect multiple levels of severity. Performance was evaluated based on the results reported in 12 studies.

### 5.1. Main Findings

The detection and classification of Parkinson’s disease severity through gait analysis demonstrated high accuracy, with an overall pooled value of 96.4% (95% CI: 95.9–96.9%). This performance was very high across all H&Y levels (ranging from 95.9% to 97.3%). All proposed methods demonstrated a strong ability to detect true negatives (specificity between 97.1% and 98.2% with an overall pooled value of 97.7%) and to predict true positives (precision between 92.0% and 95.1% with an overall pooled value of 93.4%). However, the more advanced the symptoms, the less effective the detection of true positives (sensitivity: 97.3% for H&Y0 vs. 92.1% for H&Y3, Table 4), which impacts the F1 score. Subgroup analysis using AI methods revealed differing results. Machine learning (ML) models achieved the best overall results for identifying healthy subjects (H&Y0, performance ranging from 95.7% to 98.2%), while deep learning (DL) approaches outperformed ML approaches in classifying the different severity levels of Parkinson’s patients (performance exceeding 92% for all performance metrics).

### 5.2. Interpretation in Light of the Existing Literature

In AI, performance analysis is an essential criterion for the viability of a diagnostic aid method. Systematic reviews and meta-analyses evaluating the performance of AI methods have been conducted in several areas, such as fall detection in the elderly [68], pancreatic cancer [69], cervical cancer [70], and the prevention of work-related musculoskeletal disorders [71]. In the context of Parkinson’s disease detection, few meta-analyses have been conducted on performance. Wang et al. [72] performed an analysis of sensitivity and specificity by combining the detection performance for ML and DL from positron emission tomography imaging. The authors reported performances of 87.84% and 84.69% for DL and 79.44% and 83.05% for ML, respectively, for these two parameters. Twala [73] proposed a meta-analysis of AI methods performance for detecting PD patients and classifying severity according to MDS-UPDRS Part III, considering a multimodal approach: analysis of motor symptoms, voice pattern recognition, and gait analysis. The author reported that gait assessment achieved 91.7% accuracy. Other studies addressing performance are mainly surveys and, more rarely, systematic reviews whose objective is to provide an overview by breaking down the datasets, methods, and algorithms used [7,74,75,76]. None of these solutions quantify the performance of the different methods through rigorous statistical analysis.

Beyond systematic analyses, there are several methods and algorithms that have shown high performance in detecting and classifying the severity of Parkinson’s disease. di Biase et al. [41] reported in a systematic review the best accuracy of ML algorithms (KNN: 99.4%, NB: 92.2%, LR: 81.0%, SVM: 99.9%, DT: 95.0%, RF: 100%, ANN: 96.3%) on gait analysis data from wearable sensors. Torghabeh et al. [37] proposed a DL approach integrating an LSTM and a bi-LSTM, which achieved performance greater than 99% for sensitivity, specificity, precision, and F1-score (accuracy was 92%). Archila et al. [39] achieved 100% performance on all parameters with a CNN (DL) by combining the features of fusing eye and gait motion patterns. Lin et al. [77] also proposed a DL approach based on a CNN and achieved performance greater than 99% for all five parameters. By integrating severity classification, Ji et al. [78] used a spatial-temporal CNN-transformer model for PD severity classification and achieved performance between 98 and 100% for the five parameters, considering four levels of H&Y score (0, 2, 2.5, 3). Similarly, Vidya et al. [66] proposed a DL solution based on a CNN-LSTM to classify patients according to the same four H&Y levels. The authors reported performance ranging from 97% to 100% for all parameters. However, these studies do not use the same datasets, which makes comparison between them difficult. Indeed, it has been shown that the dataset has a major impact on the performance values of an AI method [42,71]. Althnian et al. [79] demonstrated through an analysis including six ML classifiers and 20 datasets from the medical field that accuracy was affected by the size of the dataset.

By considering only a single dataset and performing a meta-analysis, the present study made it possible to compare the different ML and DL methods for detecting and classifying PD patients.

### 5.3. Clinical Relevance of Study Findings

The H&Y scale is the clinical reference tool for describing how the symptoms of Parkinson’s disease progress and allows patients to be classified according to their degree of autonomy. However, in some cases it can be difficult to classify a patient whose motor skills are close to the boundary between two levels. This depends on the experience and subjectivity of the evaluators, which can lead to some variability in the classification. There is also intra-individual variability in the expression or progression of the disease’s symptoms, which can manifest as subtle motor changes that may be difficult to detect, thus leading to differences in classification [41]. Finally, there is intra-patient variability related to daily fluctuations in the motor and non-motor symptoms, which can also lead to differences in classification [80,81]. Consequently, the use of AI-based automated methods is not intended to replace experts, but is a good way to complement their diagnosis and assist in decision-making.

Assessing gait is a relevant approach for monitoring disease progression, since walking difficulties appear as body rigidity and postural instability increase [82]. In this study, the diagnostic aid is based on the use of a wearable system consisting of insoles with plantar pressure sensors. There are many advantages to this type of device: they are easy to use, inexpensive, and technological advances are making them increasingly robust. They allow for continuous monitoring and can therefore be used to track daily fluctuations in motor symptoms related to walking [83]. The use of vertical ground reaction forces has shown promising results for classification and detection with an accuracy ranging from 94.9% to 97.1%. However, the results showed lower performance in the assessment of stage 3 of the H&Y scale. Further research and development of AI methods is necessary to improve their performance and extend it to all levels (from 0 to 5). Due to the multifactorial nature of Parkinson’s disease, one approach would be to consider a multimodal approach integrating other aspects such as tremor, speech, or voice patterns [73,84].

Another important aspect to consider is the fact that AI methods require a training phase using a specific dataset that determines their performance. It is necessary to ensure that a wide variety of situations are included during training, or to retrain the model with new labeled data to ensure effective detection during clinical examination [26,79].

### 5.4. Quality Assessment and Heterogeneity of PD Classification Devices

Full adherence to the PRISMA guidelines identified areas for methodological improvement in the included studies. The main improvement concerns the risk of bias highlighted by the PROBAST assessment. Assessing risk of bias is essential for evaluating the validity of the reported performance data. It helps determine whether variations are due to methodological weaknesses or to actual differences in the performance of the proposed solutions [85]. In the present meta-analysis, all included studies presented a “high” risk of bias related to methodological weaknesses. Although the predictors and outcomes were correctly defined, the studies rely on a dataset that is limited in the number of subjects and unbalanced across classes, which partially explains the observed publication biases. Moreover, the studies never performed external validation, which increases the likelihood of overfitting and optimistic performance estimates. Despite this finding, the meta-analysis revealed that the results remained robust, as the sensitivity analysis showed a variation in performance metrics of less than 1.5%. This step remains important because it allows us to determine whether a particular study or criterion could lead to a significant variation in the results obtained. This was not the case in the present analysis, as the small variations reflect good performance stability. Therefore, in the future, the PhysioNet dataset should be expanded by incorporating more subjects while balancing the severity classes, and the proposed models should be validated on a second external dataset to improve the generalizability of Parkinson’s disease classification systems.

The second important point concerns the heterogeneity of the results, which was high for accuracy, sensitivity, and specificity. With an I^2^ > 75% and a τ^2^ > 0.6, significant inter-study variability was observed, this is contrary to the expected results, i.e., a reduction in heterogeneity by limiting the selection of studies to a single dataset. Indeed, there is clinical (dataset) and methodological diversity among studies, which is a major source of heterogeneity in meta-analyses [55]. Reducing this diversity by selecting highly homogeneous studies is a classic strategy for reducing heterogeneity. However, this hypothesis was not verified by restricting the analysis to the PhysioNet dataset alone. In the future, it seems more appropriate to consider it and attempt to explain it rather than artificially eliminate this heterogeneity [86]. This was the objective of the in-depth analyses. However, despite a subgroup analysis conducted between ML and DL approaches, the slight reduction in I^2^ and τ^2^ was insufficient, and heterogeneity remained high. A significant reduction in this heterogeneity was observed when only the best AI method from each included study was selected. Considering all the solutions could therefore explain part of the observed heterogeneity (particularly due to a lack of statistical independence in the data). However, despite this reduction, a large proportion of the I^2^ values remained above 50%, indicating that heterogeneity remains high. This suggests that numerous other methodological factors are responsible for the differences observed between studies. On the one hand, the choice of features used as input to the model can affect the measured performance. Balaji et al. [61] demonstrated a performance difference between kinematic and statistical features for their AI model using an SVM. The authors observed a difference of approximately 2% for healthy versus Parkinson’s classification in terms of accuracy, sensitivity, and F1 score, reaching 8% for sensitivity in the H&Y2.5 class. Data windowing also affects performance levels. Another study by Balaji et al. [63] highlighted a difference when the size of the data window for vertical reaction forces varied (300 frames vs. 500 frames). Furthermore, regardless of the features, the characteristics of the classifiers also affect performance. Studies have highlighted differences between various classifiers, whether they are ML [87] or DL [21]. Vidya et al. [65] also demonstrated this effect by testing different functions of an SVM classifier (linear, Gaussian, cubic, quadratic). It is therefore important to continue in-depth analyses, particularly by pooling together studies that share the same methodological characteristics in order to assess the effect of each of these parameters on classification performance.

All of these factors contribute to the level of confidence placed in a meta-analysis, which is essential for interpreting the results and their potential applications [88]. The higher the level of evidence, the more the results can be used to make decisions and formulate recommendations. Conversely, a low degree of certainty calls for caution and indicates the need for further studies to confirm the results. In the context of this meta-analysis, the level of evidence was judged to be low for precision and F1 score and very low for accuracy, sensitivity, and specificity. Then, it is difficult to generalize the results. Thus, it is crucial that future primary studies use rigorous methodological tools such as PROBAST to ensure the quality of performance results, their use in real-world settings, and their application in research.

### 5.5. Limitations

The first limitation concerns the choice of a single dataset. Although this standardized the comparison of AI methods, this choice limits the generalization of performance results, since an algorithm trained on one dataset generally performs significantly worse on another.

The second limitation concerns the number and diversity of existing solutions in the literature. Only 12 studies were included, and despite the 176 different algorithms, several of them were very poorly represented (only once for some). Moreover, two-thirds of the studies come from the same country (India), which could introduce systematic bias in the design of algorithms and validation choices. These different elements limited the statistical power and therefore the generalization of the results.

The third limitation concerns the significant heterogeneity and inter-study variance observed. Despite the subgroup analyses (ML vs. DL and only the best AI method for each study) and a reduction in heterogeneity, the latter remained high. Other factors not taken into account, such as treatment techniques, the classifiers used, or the number and nature of the AI model’s input data, may influence the results.

The fourth limitation concerns the low level of evidence in the results, primarily due to the high risk of bias in all included studies. Consequently, the conclusions should be interpreted with caution, and further analyses are needed to generalize the presented results. The application of methodological recommendations from diagnostic test accuracy studies, such as PROBAST, appears essential for using these results in future research.

Our study focused on the PD diagnosis based on gait analysis, excluding other symptoms such as tremors, speech, or writing impairments. This inevitably limited the number of studies considered.

Finally, methodological limitations, particularly those related to the criteria for selecting articles, should be mentioned. The choice of databases, the combination of specific keywords without necessarily using all synonyms, and the restriction to original, peer-reviewed studies written in English may have led to the omission of some relevant studies.

### 5.6. General Outcomes and Future Research Directions

This systematic review only considered studies using the Gait in Parkinson’s Disease dataset containing 93 PD patients and 73 healthy control subjects classified according to the H&Y scale. However, there are other public datasets (Smart-Insole dataset [89] or Herbers Insole Pressure dataset [90]) or customized datasets [91] that include gait data from PD subjects. Given that a model depends on its training dataset, it would be useful to integrate all this data into a single reference dataset in order to test the performance of AI models in a standardized setting that includes the widest range of gait symptoms in PD patients. This increase in the number of subjects would help reduce the risk of bias (particularly in PROBAST domain 1), enable external validation, which is still lacking in many studies, and ensure its effective transferability to the clinical field.

The integration of AI methods with wearable sensors represents a significant step forward toward accurate and comprehensive classification of disease stages. Based on continuous monitoring, this technology provides personalized feedback for each patient, which can be used to individualize treatment according to symptom fluctuations. One of the objectives is to develop an accurate and automated closed-loop system for the diagnosis and management of Parkinson’s disease.

To achieve this goal, it is necessary to consider the various constraints associated with the use of wearable sensors. System autonomy, usability (few sensors and quick and easy setup), and acceptability are essential for a viable and effective wearable system. Finally, the development of secure data storage and transmission methods is essential for protecting users’ privacy.

Ultimately, the classification of gait data using AI methods could be integrated into multifactorial detection systems that incorporate other aspects of Parkinson’s disease to regulate or adapt treatment based on the patient’s real-time motor status [92]. Thus, future AI detection models could enable self-monitoring, and the data could be integrated and combined with healthcare professionals’ diagnoses to improve the care of PD patients. These advances could also be used for the early detection of motor impairment, continuous monitoring of functional changes, and tracking of loss of autonomy in other neurodegenerative diseases such as Alzheimer’s disease, Huntington’s disease, and amyotrophic lateral sclerosis.

## 6. Conclusions

This literature review provided an overview of the performance of systems for detecting and classifying PD patients based on symptom severity using a single dataset in accordance with PRISMA guidelines. ML algorithms achieved high accuracy in binary classification—healthy individuals versus PD patients—while DL models performed better in classifying advanced stages of the disease. However, the reported results are likely optimistic and probably overestimated, given the methodological weaknesses resulting in a high risk of bias and insufficient level of evidence. Further research using a rigorous methodology (PROBAST) is needed to ensure the generalizability of the results and the effective clinical transferability of the proposed solutions.

## Figures and Tables

**Table 1:** Demographics of healthy subjects and PD patients included in Gait in Parkinson’s Disease dataset detailed by Ga, Ju, Si dataset.

Dataset	Group	Subjects	H&Y Scale Score (*n*)	Male	Female	Age		Height (m)	Weight (kg)
						Mean ± SD	Range		
Ga [34]	Healthy	18	0 (18)	10	8	57.9 ± 6.7	37–70	1.68 ± 0.08	74.2 ± 12.7
	PD	29	2 (15); 2.5 (8); 3 (6)	20	9	61.6 ± 8.8	36–77	1.67 ± 0.07	73.1 ± 11.2
Ju [35]	Healthy	26	0 (26)	12	14	39.31 ± 18.51	20–74	1.83 ± 0.08	66.8 ± 11.07
	PD	29	2 (12); 2.5 (13); 3 (4)	16	13	66.80 ± 10.85	44–80	1.87 ± 0.15	75.1 ± 16.89
Si [36]	Healthy	29	0 (29)	18	11	64.5 ± 6.8	53–77	1.69 ± 0.08	71.5 ± 11.0
	PD	35	2 (29); 2.5 (6); 3 (0)	22	13	67.2 ± 9.1	61–84	1.66 ± 0.07	70.3 ± 8.4

H&Y scale: Hoehn and Yahr scale; PD: Parkinson disease; SD: standard deviation.

**Table 2:** Keyword combination for each database.

Database	Keyword Combinations
PubMed/Medline Google Scholar IEEE Xplore	(PhysioNet) AND (“Parkinson’s disease”) AND detection AND (“Gait analysis”) AND (“Deep learning” OR “Machine learning”) AND (Accuracy) AND (Hoehn OR HY OR “H&Y”)
ScienceDirect	(PhysioNet) AND (“Parkinson’s disease“) AND detection AND (“Gait analysis”) AND (“Deep learning” OR “Machine learning”) AND (Accuracy) AND (Hoehn OR “H&Y”)

**Table 3:** Detailed study characteristics, AI methods and algorithms tested and performances parameters used in the 12 included studies.

Authors	Year	Country	Method	Algorithms	Number of Methods Tested	Accuracy	Specificity	Sensitivity	Precision	F1-Score
Balaji et al. [61]	2020	India	ML	DT, SVM, EC, BC	32	X	X	X		
Balaji et al. [14]	2021	India	ML	KNN, EC, NB, SVM	16	X	X	X	X	X
Balaji et al. [62]	2021	India	DL	LSTM	8	X	X	X		X
Balaji et al. [63]	2024	India	DL	CNN	16	X	X	X	X	X
Balakrishnan et al. [64]	2023	India	ML	RF	4	X	X	X	X	X
Huan et al. [67]	2025	USA	DL	GRU	4	X		X	X	X
Mittal et al. [32]	2025	India	ML	DT, RF, XGBoost, LightGBM	16	X		X	X	X
Naimi et al. [58]	2024	Canada	DL	CNN	4			X	X	X
Vidya et al. [65]	2021	India	ML	SVM	44	X	X	X		
Vidya et al. [66]	2022	India	DL	CNN, LSTM	8	X	X	X	X	X
Wang et al. [59]	2024	China	ML	SVM, KNN, NB, DT, AdaBoost	20	X	X	X		X
Zhao et al. [60]	2022	China	ML	KNN	4			X	X	X

DL = Deep Learning; ML = Machine Learning; DT = Decision Tree; AdaBoost = Adaptive Boosting; BC = Bayes Classifier; CNN = Convolutional Neural Networks; EC = Ensemble classifier; GRU = Gated Recurrent Unit; KNN = K-Nearest Neighbors; LightGBM = Light Gradient-Boosting Machine; LSTM = Long Short-Term Memory; NB = Naïve Bayes Classifier; RF = Random Forest; SVM = Support Vector Machine; XGBoost = eXtreme Gradient Boosting. X indicates that the authors reported a performance value for this parameter.

**Table 4:** Results of overall performance and by class H&Y.

H&Y	Statistics	Accuracy	Specificity	Sensitivity	Precision	F1 Score
All classes	Nb algo	165	148	176	80	100
	Pooled value	96.4%	97.7%	94.0%	93.4%	92.1%
	95% CI Lower	95.9%	97.3%	92.7%	92.0%	90.6%
	95% CI Upper	96.9%	98.1%	95.2%	94.6%	93.4%
	τ^2^	0.60	0.97	1.40	0.28	0.38
	I^2^	78.67%	77.25%	85.46%	39.63%	49.62%
H&Y0	Nb algo	43	38	45	20	25
	Pooled value	97.3%	97.7%	97.3%	95.1%	94.9%
	95% CI Lower	96.1%	96.7%	96.2%	92.5%	93.2%
	95% CI Upper	98.1%	98.4%	98.1%	96.8%	96.2%
	τ^2^	0.69	0.59	0.66	0.41	0.16
	I^2^	76.13%	60.49%	67.22%	55.50%	33.03%
H&Y2	Nb algo	42	38	45	20	25
	Pooled value	95.9%	97.1%	92.9%	92.1%	91.5%
	95% CI Lower	94.5%	95.6%	91.1%	89.1%	89.2%
	95% CI Upper	96.9%	98.1%	94.4%	94.4%	93.5%
	τ^2^	0.60	1.12	0.35	0.25	0.18
	I^2^	81.40%	80.98%	53.64%	48.22%	40.51%
H&Y2.5	Nb algo	42	38	45	20	25
	Pooled value	95.9%	97.8%	90.0%	93.2%	90.4%
	95% CI Lower	94.4%	96.8%	85.6%	90.6%	87.1%
	95% CI Upper	97.0%	98.4%	93.2%	95.2%	92.9%
	τ^2^	0.71	0.77	1.28	0	0.11
	I^2^	83.92%	71.74%	73.49%	0%	19.22%
H&Y3	Nb algo	38	34	41	20	25
	Pooled value	96.7%	98.2%	92.1%	92.0%	85.8%
	95% CI Lower	95.6%	97.1%	86.1%	86.1%	77.7%
	95% CI Upper	97.5%	98.8%	95.6%	95.5%	91.4%
	τ^2^	0.32	1.31	2.87	0.64	1.00
	I^2^	61.42%	83.27%	95.94%	37.21%	54.18%

Nb algo: number of available data used; CI: confidence interval.

**Table 5:** Performance difference between estimates pooled from all available AI methods and pooled only from the best method of each included study by class H&Y.

H&Y	Statistics	Accuracy	Specificity	Sensitivity	Precision	F1 Score
All classes	Nb algo	−128	−116	−128	−44	−60
	Pooled value	1.64%	0.76%	0.48%	1.08%	1.60%
	95% CI Lower	1.41%	0.60%	−0.15%	0.41%	1.08%
	95% CI Upper	1.67%	0.78%	0.57%	1.41%	1.81%
	τ^2^	−0.11	−0.7	−0.62	0.21	−0.07
	I^2^	−18.00%	−45.00%	−7.18%	13.53%	−8.78%
H&Y0	Nb algo	−33	−30	−33	−11	−15
	Pooled value	0.49%	0.26%	−0.41%	0.91%	1.18%
	95% CI Lower	−0.93%	−0.02%	−1.67%	−2.03%	−0.79%
	95% CI Upper	0.90%	0.35%	0.17%	1.59%	1.82%
	τ^2^	0.09	−0.59	−0.25	0.6	0.39
	I^2^	−3.13%	−60.49%	−10.50%	18.46%	25.10%
H&Y2	Nb algo	−33	−30	−33	−11	−15
	Pooled value	1.49%	0.58%	1.09%	0.45%	1.46%
	95% CI Lower	0.89%	−0.48%	−0.90%	−3.41%	−1.55%
	95% CI Upper	1.68%	0.81%	1.94%	1.83%	2.55%
	τ^2^	−0.32	−0.86	0.01	0.16	0.19
	I^2^	−29.15%	−47.22%	−3.00%	9.25%	13.00%
H&Y2.5	Nb algo	−33	−30	−33	−11	−15
	Pooled value	2.40%	0.54%	1.45%	−0.05%	1.28%
	95% CI Lower	1.90%	−0.09%	1.16%	−3.28%	−0.69%
	95% CI Upper	2.23%	0.77%	1.43%	1.24%	2.17%
	τ^2^	−0.09	−0.52	−1.28	0.05	−0.11
	I^2^	−22.80%	−39.00%	−73.49%	7.00%	−19.22%
H&Y3	Nb algo	−29	−26	−29	−11	−15
	Pooled value	1.95%	1.15%	0.31%	3.97%	6.96%
	95% CI Lower	1.96%	1.81%	−2.35%	6.17%	8.36%
	95% CI Upper	1.75%	0.81%	1.01%	2.42%	4.97%
	τ^2^	−0.18	−1.31	−1.46	−0.64	−0.82
	I^2^	−42.17%	−83.27%	−2.55%	−37.21%	−38.24%

Nb algo: number of available data used; CI: confidence interval. Pooled estimates from all available AI methods were used as a reference for computing the difference.

**Table 6:** ML and DL methods performance comparison for each level of the H&Y scale.

H&Y	Statistics	Accuracy		Specificity		Sensitivity		Precision		F1-Score	
		ML	DL	ML	DL	ML	DL	ML	DL	ML	DL
H&Y0	Nb algo	34	9	30	8	35	10	10	10	15	10
	Pooled value	97.9%	96.0%	98.2%	96.4%	98.1%	95.4%	97.3%	93.5%	95.7%	94.0%
	95% CI Lower	96.6%	93.5%	97.2%	95.0%	97.0%	93.0%	93.9%	89.5%	93.2%	91.1%
	95% CI Upper	98.6%	97.6%	98.9%	97.4%	98.8%	97.0%	98.8%	95.9%	97.3%	96.1%
	τ^2^	1.03	0.33	0.95	0	1.06	0.18	0.80	0.31	0.20	0.16
	I^2^	81.00%	66.75%	67.45%	0%	70.77%	50.21%	58.52%	56.59%	33.36%	38.31%
H&Y2	Nb algo	34	8	30	8	35	10	10	10	15	10
	Pooled value	95.6%	96.7%	97.0%	97.4%	92.9%	93.1%	90.1%	93.9%	90.6%	93.2%
	95% CI Lower	93.9%	95.2%	95.0%	95.9%	90.6%	89.4%	84.8%	89.8%	87.1%	89.2%
	95% CI Upper	96.9%	97.8%	98.2%	98.4%	98.0%	95.5%	93.8%	96.4%	93.2%	95.9%
	τ^2^	0.70	0.05	1.40	0	0.41	0.16	0.12	0.36	0.16	0.25
	I^2^	84.54%	18.92%	85.25%	0%	57.60%	34.68%	33.57%	51.53%	39.40%	43.42%
H&Y2.5	Nb algo	34	8	30	8	35	10	10	10	15	10
	Pooled value	95.2%	97.7%	97.4%	98.7%	89.0%	92.5%	93.1%	93.5%	88.9%	92.3%
	95% CI Lower	93.3%	96.4%	96.0%	97.7%	82.8%	88.4%	88.7%	88.6%	83.5%	88.2%
	95% CI Upper	96.6%	98.6%	98.3%	99.2%	93.2%	95.2%	95.8%	96.4%	92.9%	95.1%
	τ^2^	0.68	0.06	0.76	0	1.58	0	0	0.05	0.12	0
	I^2^	84.97%	16.82%	73.74%	0%	77.94%	0%	0%	7.65%	22.33%	0%
H&Y3	Nb algo	30	8	26	8	31	10	10	10	15	10
	Pooled value	96.0%	98.2%	97.5%	99.3%	91.8%	92.9%	85.8%	95.6%	77.4%	93.6%
	95% CI Lower	94.8%	96.7%	95.8%	98.8%	82.8%	84.9%	67.5%	92.9%	62.7%	88.3%
	95% CI Upper	97.0%	99.0%	98.5%	99.6%	96.3%	96.6%	94.7%	97.3%	87.3%	96.6%
	τ^2^	0.22	0.17	1.15	0	3.95	0.98	1.16	0	0.77	0
	I^2^	55.24%	29.51%	83.79%	0%	96.71%	90.04%	52.11%	0%	50.29%	0%

95% CI: 95% confidence interval; ML: machine learning; DL: deep learning; Nb algo: number of algorithms included in the analysis.

**Table 7:** Sensitivity analysis for each performance metric in relation to overall performance.

Statistics	Accuracy		Specificity		Sensitivity		Precision		F1-Score	
	Min	Max	Min	Max	Min	Max	Min	Max	Min	Max
τ^2^	0.36	0.74	0.72	1.21	1.02	1.6	0.2	0.36	0.28	0.49
I^2^	68.50%	79.95%	70.03%	80.26%	82.70%	86.71%	32.16%	46.16%	42.43%	56.11%
Pooled value	96.16%	96.77%	97.63%	98.04%	92.62%	94.88%	93.02%	93.70%	91.45%	93.15%
Maximum difference with overall	0.33%		0.29%		1.43%		0.38%		1.09%	

**Table 8:** Quality of evidence description based on the Grading of Recommendations, Assessment, Development and Evaluation system (GRADE).

Performance Parameter	Number of Studies	Certainty Assessment						Effect			Overall Level of Evidence
		Study Design	Publication Bias (Egger Test)	Indirectness ^a^	Inconsistency ^b^	Imprecision ^c^	Risk of Bias ^d^	*n*	Event Rate	(95% CI)	
Accuracy	10	DTAS	Serious (*p* < 0.05)	Not serious	Serious (I^2^ = 78.67%)	Not serious	Serious	165	96.4%	95.9–96.9%	Very low ⬤◯◯◯
Sensitivity	12	DTAS	Serious (*p* < 0.05)	Not serious	Serious (I^2^ = 85.46%)	Not serious	Serious	176	94.0%	92.7–95.2%	Very low ⬤◯◯◯
Specificity	8	DTAS	Serious (*p* < 0.05)	Not serious	Serious (I^2^ = 77.25%)	Not serious	Serious	148	97.7%	97.3–98.1%	Very low ⬤◯◯◯
Precision	8	DTAS	Serious (*p* < 0.05)	Not serious	Not serious (I^2^ = 39.63%)	Not serious	Serious	80	93.4%	92.0–94.6%	Low ⬤⬤◯◯
F1-score	10	DTAS	Serious (*p* < 0.05)	Not serious	Not serious (I^2^ = 49.62%)	Not serious	Serious	100	92.1%	90.6–93.4%	Low ⬤⬤◯◯

DTAS: Diagnostic test accuracy study; *n*: number of available data; 95% CI: 95% confidence interval; GRADE overall quality significance: ⬤◯◯◯ = Very low; ⬤⬤◯◯ = Low; ⬤⬤⬤◯ = Moderate; ⬤⬤⬤⬤ = High. ^a^ Studied population correspond to the population in study; ^b^ Serious if I^2^ > 50%; ^c^ Serious if 95% CI Range Difference > 50% of Event Rate; ^d^ Studies have at least a fair critical appraisal score.

## Data Availability

The original contributions presented in this study are included in the article. Further inquiries can be directed to the corresponding author.

## Supplementary Materials

The following supporting information can be downloaded at: https://www.mdpi.com/article/10.3390/healthcare14131820/s1, the five Supplementary Files (Figures S1–S5) attached to this article contain detailed forest plots of each performance parameter by severity level of Parkinson’s disease. Figure S1: Forest plot of logit transformed accuracy pooled across studies by H&Y score. Each square represents the logit transformed accuracy of one algorithm. The horizontal bars represent the 95% confidence interval. The diamonds at the bottom represent the pooled accuracy by H&Y score. The last diamond represents the overall pooled accuracy across all studies. References [14,32,59,61,62,63,64,65,66,67]. Figure S2: Forest plot of logit transformed specificity pooled across studies by H&Y score. Each square represents the logit transformed specificity of one algorithm. The horizontal bars represent the 95% confidence interval. The diamonds at the bottom represent the pooled specificity by H&Y score. The last diamond represents the overall pooled specificity across all studies. References [14,59,61,62,63,64,65,66]. Figure S3: Forest plot of logit transformed sensitivity pooled across studies by H&Y score. Each square represents the logit transformed sensitivity of one algorithm. The horizontal bars represent the 95% confidence interval. The diamonds at the bottom represent the pooled sensitivity by H&Y score. The last diamond represents the overall pooled sensitivity across all studies. References [14,32,58,59,60,61,62,63,64,65,66,67]. Figure S4: Forest plot of logit transformed precision pooled across studies by H&Y score. Each square represents the logit transformed precision of one algorithm. The horizontal bars represent the 95% confidence interval. The diamonds at the bottom represent the pooled precision by H&Y score. The last diamond represents the overall pooled precision across all studies. References [14,32,58,60,63,64,66,67]. Figure S5: Forest plot of logit transformed F1 score pooled across studies by H&Y score. Each square represents the logit transformed F1 score of one algorithm. The horizontal bars represent the 95% confidence interval. The diamonds at the bottom represent the pooled F1 score by H&Y score. The last diamond represents the overall pooled F1 score across all studies. References [14,32,58,59,60,62,63,64,66,67].

## Abbreviations

The following abbreviations are used in this manuscript:

AdaBoost	Adaptive Boosting
AI	Artificial Intelligence
BC	Bayes Classifier
CNN	Convolutional Neural Networks
DL	Deep Learning
DT	Decision Tree
EC	Ensemble classifier
EMG	Eletromyography
FN	False Negative
FP	False Positive
GRADE	Grading of Recommendations Assessment, Development, and Evaluation
GRU	Gated Recurrent Unit
H&Y scale	Hoehn and Yahr scale
IMU	Inertial Measurement Unit
KNN	K-Nearest Neighbors
LightGBM	Light Gradient-Boosting Ma-chine
LSTM	Long Short-Term Memory
MDS-UPDRS	Movement Disorder Society’s Unified Parkinson’s Disease Rating Scale
ML	Machine Learning
NB	Naïve Bayes Classifier
PD	Parkinson Disease
PRISMA	Preferred Reporting Items for Systematic reviews and Meta-Analyses
PROBAST	Prediction Model Study Risk of Bias Assessment Tool
RF	Random Forest
SD	Standard deviation
SVM	Support Vector Machine
TN	True Negative
TP	True Positive
VGRF	Vertical Ground Reaction Forces
XGBoost	eXtreme Gradient Boosting.

## Appendix A. Performance Based on the Best AI Method from Each Included Study

Table A1 presents the detailed performance results obtained by considering only the best AI method presented in each of the 12 included studies. For each study, four rows were selected, one per H&Y class, containing the values of the available performance parameters. This table was used to compute the differences presented in Table 5 in Section 4.5.

**Table A1:** Performance pooled only from the best AI method of each included study by class H&Y.

H&Y	Statistics	Accuracy	Specificity	Sensitivity	Precision	F1 Score
All classes	Nb algo	10	8	12	9	10
	Pooled value	97.79%	97.96%	96.89%	96.01%	96.08%
	95% CI Lower	95.17%	96.68%	94.53%	90.47%	92.41%
	95% CI Upper	99.00%	98.75%	98.27%	98.39%	98.02%
	τ^2^	0.78	0.00	0.41	1.01	0.55
	I^2^	73.00%	0.00%	56.72%	73.96%	58.13%
H&Y0	Nb algo	9	8	12	9	10
	Pooled value	97.39%	97.68%	93.99%	92.55%	92.96%
	95% CI Lower	95.39%	95.12%	90.20%	85.69%	87.65%
	95% CI Upper	98.58%	98.91%	96.34%	96.23%	96.05%
	τ^2^	0.28	0.26	0.36	0.41	0.37
	I^2^	52.25%	33.76%	51.01%	57.47%	53.31%
H&Y2	Nb algo	9	8	12	9	10
	Pooled value	98.30%	98.34%	91.45%	93.15%	91.68%
	95% CI Lower	96.30%	96.71%	86.76%	87.32%	86.41%
	95% CI Upper	99.23%	99.17%	94.63%	96.44%	95.07%
	τ^2^	0.62	0.25	0.00	0.05	0.00
	I^2^	61.12%	32.54%	0.00%	6.94%	0.00%
H&Y2.5	Nb algo	9	8	12	9	10
	Pooled value	98.65%	99.35%	92.41%	95.97%	92.76%
	95% CI Lower	97.56%	98.91%	83.75%	92.27%	86.06%
	95% CI Upper	99.25%	99.61%	96.61%	97.92%	96.37%
	τ^2^	0.14	0.00	1.41	0.00	0.18
	I^2^	19.25%	0.00%	93.39%	0.00%	15.94%
H&Y3	Nb algo	37	32	48	36	40
	Pooled value	98.04%	98.46%	94.48%	94.48%	93.70%
	95% CI Lower	97.31%	97.90%	92.55%	92.41%	91.68%
	95% CI Upper	98.57%	98.88%	95.77%	96.01%	95.21%
	τ^2^	0.49	0.27	0.78	0.49	0.31
	I^2^	61.05%	32.64%	78.28%	53.16%	40.84%

Nb algo: number of available data used; CI: confidence interval.
