# Supplementary figures and images for: Current Trends in AI Gait Analysis for the Detection and Assessment of Parkinson’s Disease Severity: Systematic Review and Meta-Analysis of Performance Using Logit Transformation
Source: Healthcare (Basel). 2026 Jun 23;14(13):1820. doi: 10.3390/healthcare14131820 (PMC13362068; doi:10.3390/healthcare14131820)

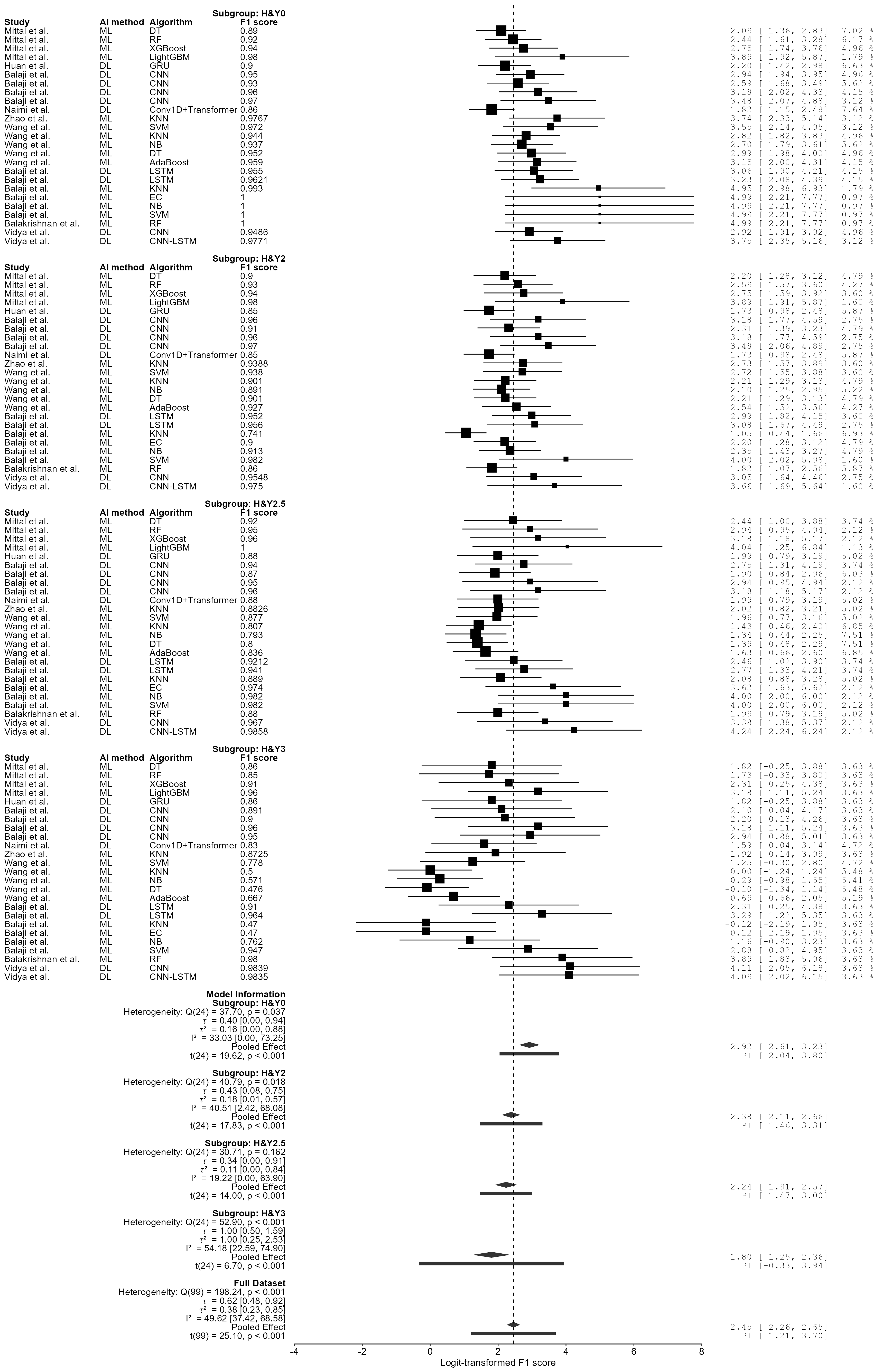

Supplement: Supplementary file 1 [file healthcare-14-01820-s001.zip › Figure S5 - Forest plot F1 score.png]

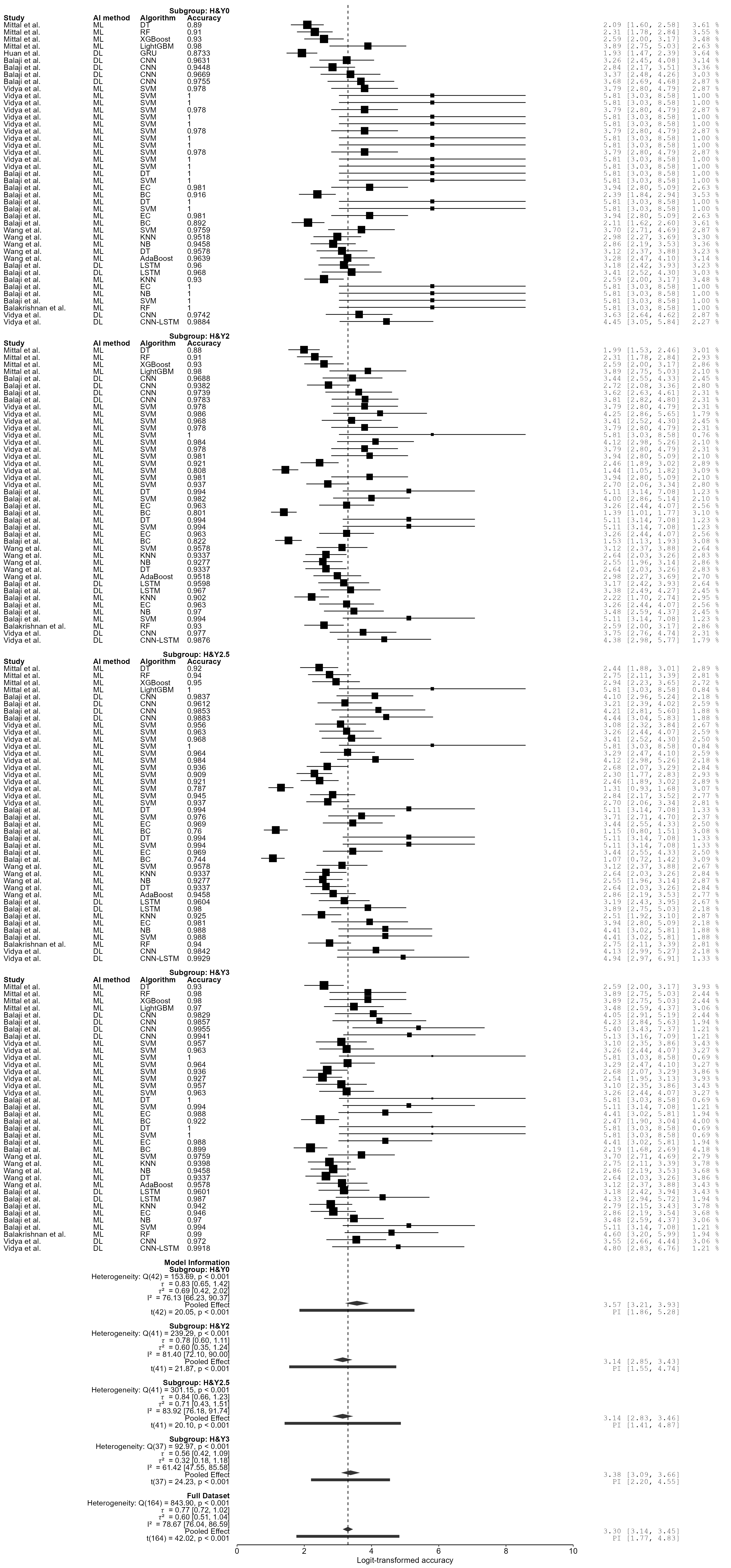

Supplement: Supplementary file 1 [file healthcare-14-01820-s001.zip › Figure S1 - Forest plot Accuracy.png]

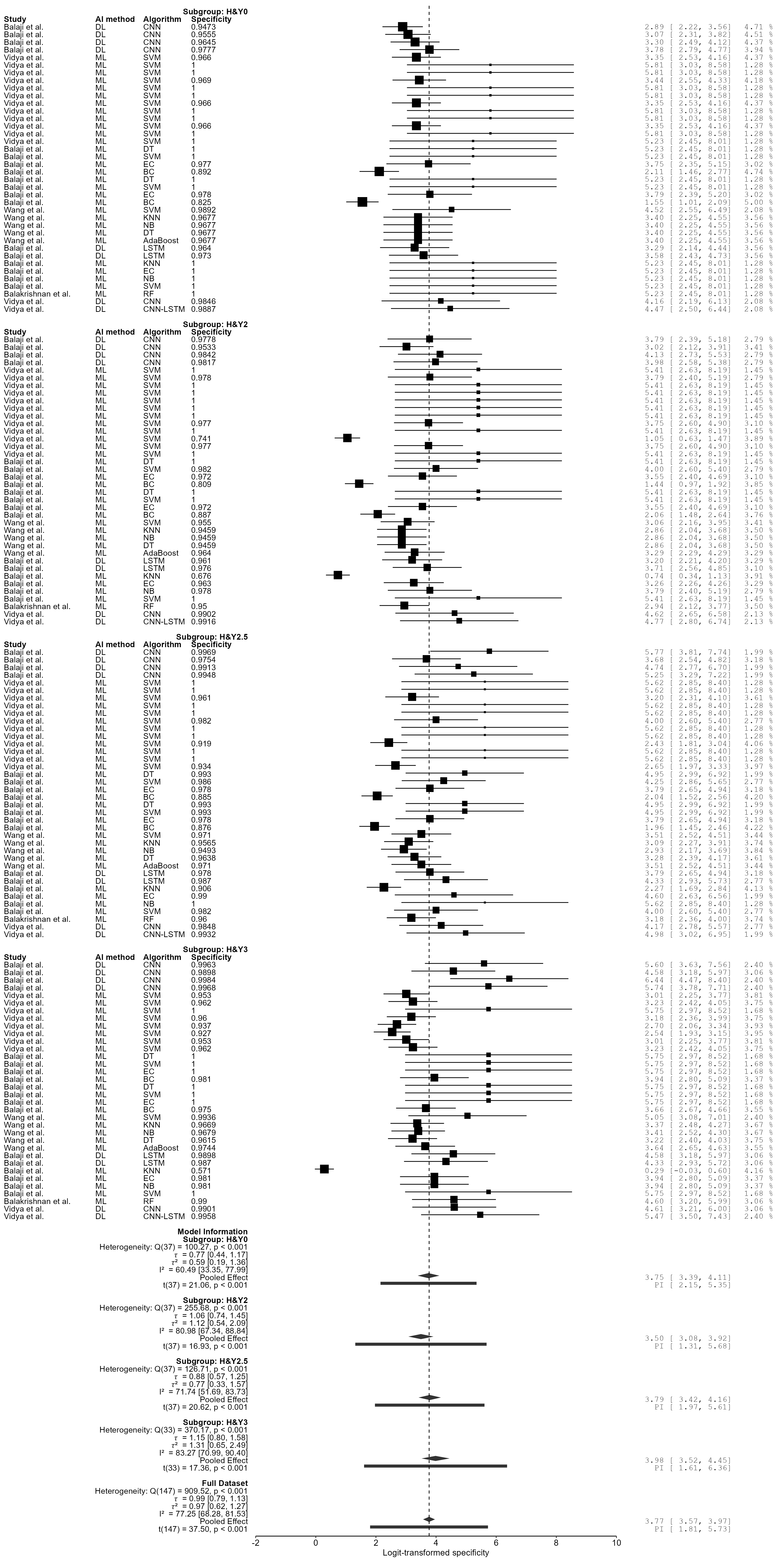

Supplement: Supplementary file 1 [file healthcare-14-01820-s001.zip › Figure S2 - Forest plot Specificity.png]

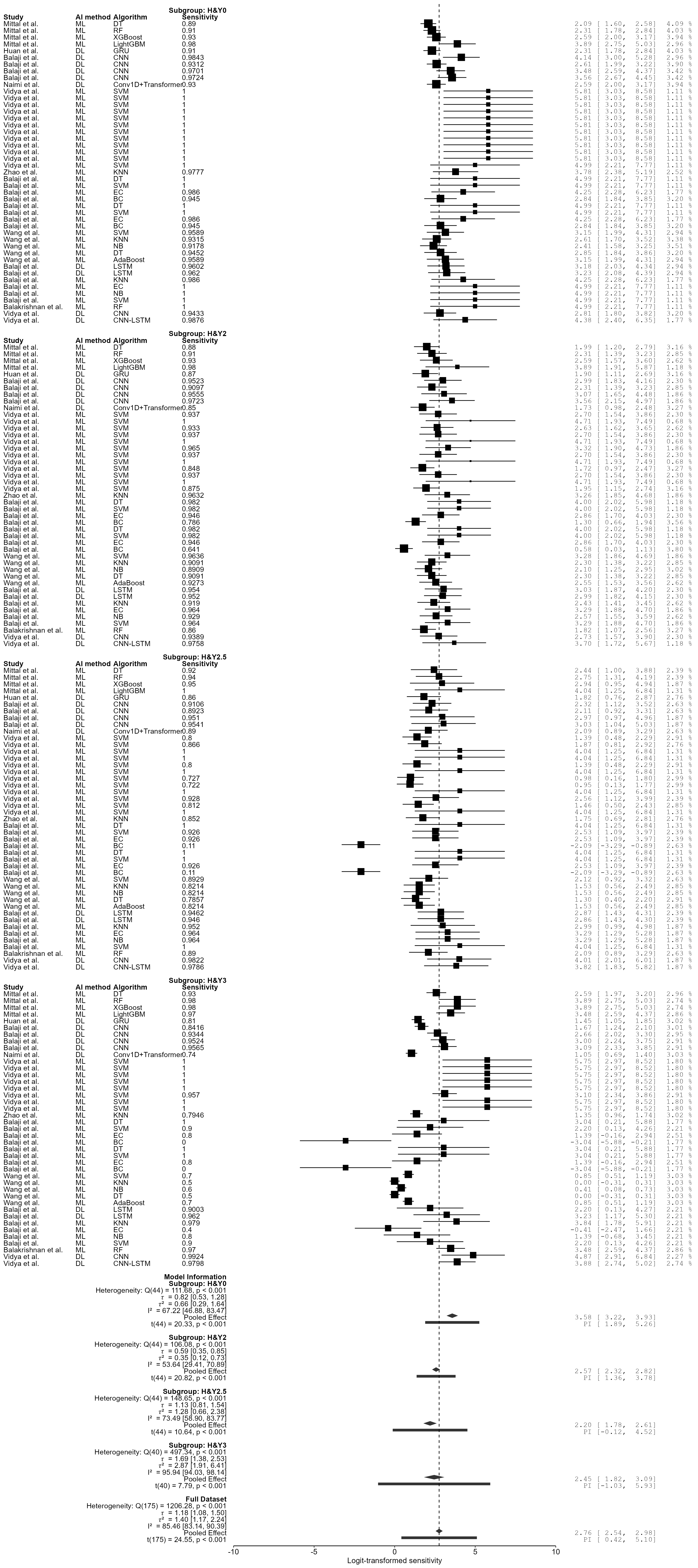

Supplement: Supplementary file 1 [file healthcare-14-01820-s001.zip › Figure S3 - Forest plot Sensitivity.png]

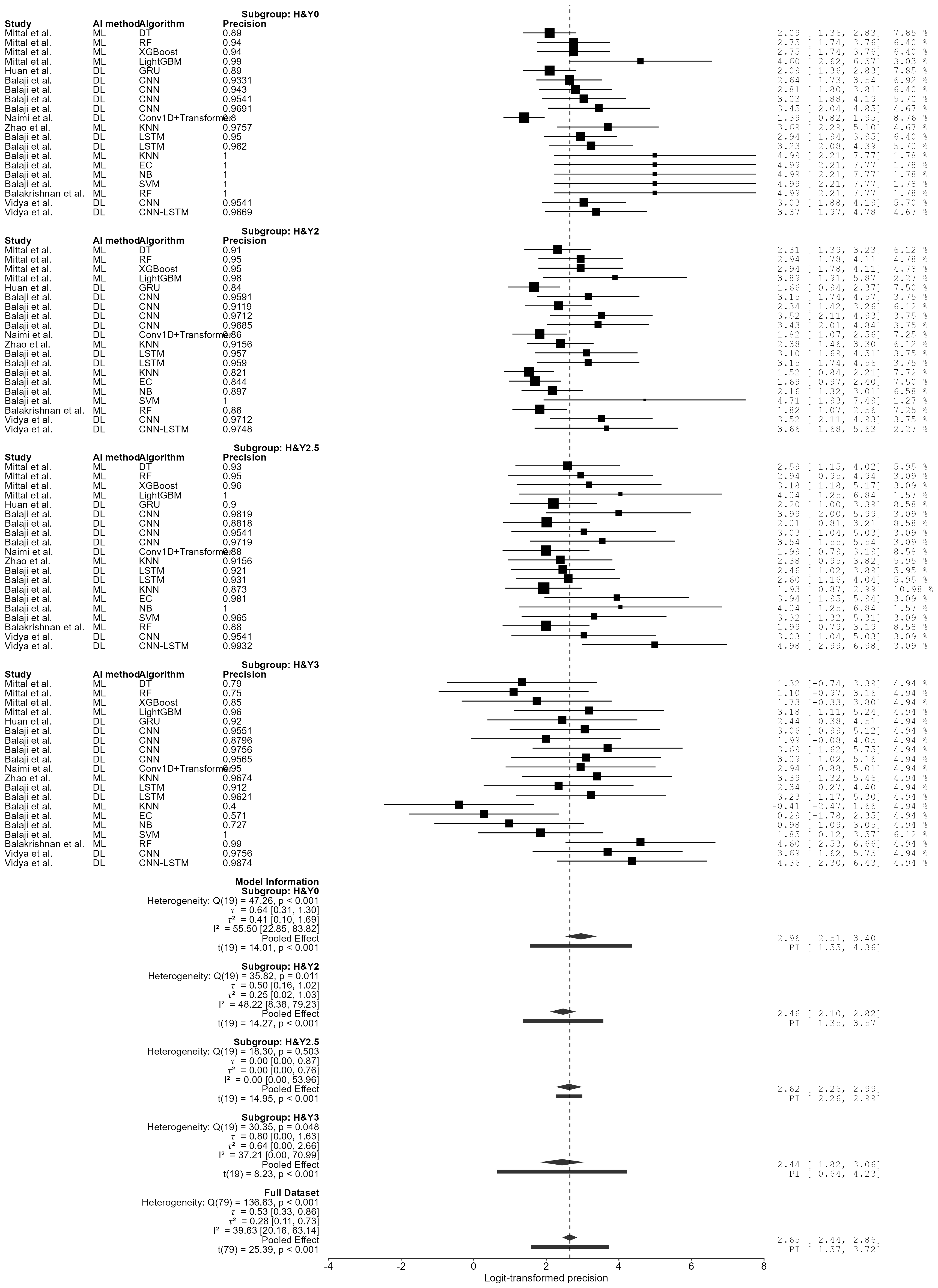

Supplement: Supplementary file 1 [file healthcare-14-01820-s001.zip › Figure S4 - Forest plot Precision.png]
